# Targeted detection of Helicobacter pylori resistance to clarithromycin and levofloxacin using single-cell Raman spectroscopy

**DOI:** 10.1099/jmm.0.002094

**Published:** 2025-12-05

**Authors:** Ziman Wu, Xinying Li, Xiaowen Dou, Haiyan Yang, Xiuming Zhang, Dan Xiong, Xiaojuan Gao

**Affiliations:** 1 School of Medical Technology, Xinxiang Medical University, Xinxiang 453003, PR China; 2 Department of Clinical Laboratory Medicine, The Third Affiliated Hospital of Shenzhen University, Shenzhen 518001, PR China; 3 School of Medicine, Anhui University of Science and Technology, Huainan 232000, PR China; 4 Shantou University Medical College, Shantou 515041, PR China

**Keywords:** clarithromycin-resistance, *Helicobacter pylori*, levofloxacin-resistance, Raman spectroscopy

## Abstract

**Introduction.** *Helicobacter pylori* infection is a major global health concern, and its increasing antibiotic resistance poses significant challenges to eradication therapy. Traditional methods for detecting *H. pylori* resistance are time-consuming and labour-intensive.

**Hypothesis/Gap Statement.** The limitations of traditional methods highlight a critical need for a rapid, accurate and comprehensive approach to detect *H. pylori* resistance that can inform personalized treatment strategies and improve eradication outcomes.

**Aim.** This study aimed to explore the potential of Raman spectroscopy as a rapid and accurate method for detecting *H. pylori* resistance to clarithromycin and levofloxacin.

**Methodology.** We employed Raman spectroscopy to analyse the metabolic fingerprints of *H. pylori* strains treated with different concentrations of antibiotics. Principal component analysis and deuterium oxide labelling techniques were used to differentiate between resistant and susceptible strains.

**Results.** Our results demonstrated that Raman spectroscopy can accurately predict *H. pylori* antibiotic resistance within 4–6 h, significantly reducing detection time compared with traditional methods.

**Conclusion.** This study provides a promising approach for rapid and accurate detection of *H. pylori* antibiotic resistance, enabling personalized treatment strategies and improving eradication outcomes.

## Introduction

*Helicobacter pylori* infection is one of the most prevalent and widespread chronic bacterial infections worldwide. Studies have shown that ~4.4 billion people are infected with *H. pylori* globally, constituting nearly half of the world’s population [1]. *H. pylori* infection plays a pivotal role in the development of various gastric disorders and significantly contributes to the progression of gastric cancer, making it a critical global public health concern [2,3]. Consequently, widespread screening and eradication programmes for *H. pylori* infection have become a public health priority in many countries. Currently, *H. pylori* eradication primarily relies on empirical treatment. However, the extensive use of empirical therapy has led to the emergence of antibiotic resistance. Globally, research has found that *H. pylori* is resistant to clarithromycin, levofloxacin and metronidazole, with resistance rates between 20% and 50% for the first two antibiotics and between 40% and 70% for the last one [4]. These resistance rates, however, vary significantly by geographic region. With increasing resistance rates, the effectiveness of empirical treatments for eradicating *H. pylori* has dropped below 80%, leading to serious challenges for therapy [5,6].

Preliminary research findings indicate that the success rate of precision therapy based on drug resistance gene testing has increased from 75% to 83.5% compared with empirical treatment. In the non-first-line treatment group, the resistance rates of levofloxacin and clarithromycin increased by 13.5% and 32.7%, respectively, compared with the initial treatment group [7]. These findings demonstrate that personalized treatment improves *H. pylori* eradication rates compared with empirical therapy. Although molecular biology methods can partially detect *H. pylori* resistance to levofloxacin and clarithromycin, there is not always complete agreement between genotype and phenotype. Additionally, suitable molecular detection techniques for other first-line antibiotics, such as metronidazole and tetracycline, are lacking. Therefore, it is essential to explore more effective methods for detecting *H. pylori* resistance to facilitate precision technique that has found extensive applications in single-cell phenotyping [8]. The use of Raman spectroscopy can guide effective treatment strategies and help reduce resistance rates. Raman microscopy is a powerful, label-free technique tool for quickly identifying bacteria. It can analyse bacterial metabolic fingerprints, offering insights into mechanisms of drug resistance and susceptibility [9,10]. Several studies have successfully used Raman spectroscopy to quickly identify antibiotic resistance profiles in important infectious micro-organisms. These include *Escherichia coli, Klebsiella pneumoniae, Streptococcus mutans, Lactobacillus fermentum, Enterococcus faecalis* and *Staphylococcus aureus* [11,13]. It has been shown that Raman spectroscopy significantly reduces detection time compared with traditional methods of isolation and identification, while maintaining result accuracy [11]. A novel method combining surface-enhanced Raman spectroscopy (SERS) and machine-learning algorithms has been developed for the accurate detection of *H. pylori* infection in human gastric juice. This method demonstrated high predictive accuracy and showed potential as a simple and rapid diagnostic tool [14]. Haider *et al*. [15] confirmed the efficacy of Raman spectroscopy in diagnosing *H. pylori* infection, finding it to be more sensitive and accurate than the rapid urease test when compared with histopathology, the gold standard. Li *et al*. [16] developed a deep learning-based method to rapidly differentiate between carcinogenic and non-carcinogenic types of *H. pylori* infection using SERS of human serum, potentially enabling early identification of individuals at high risk of gastric cancer. Sun *et al*. [17] established an 8 h rapid antimicrobial susceptibility test based on metabolomics using deuterium oxide (D_2_O)-probed Raman microspectroscopy to detect the susceptibility of *H. pylori* to metronidazole and to reveal the bacterial resistance mechanisms against metronidazole. Liu *et al*. [18] developed a novel method called Clinical Antimicrobial Susceptibility Test Ramanometry for *H. pylori* (CAST-R-HP), utilizing Raman spectroscopy, to rapidly and accurately identify *H. pylori* directly from gastric biopsy samples. This method simultaneously detects resistance to levofloxacin and clarithromycin and enables whole-genome sequencing (WGS) for in-depth analysis of resistance mechanisms [18]. In this study, we employed an Alpha300R Raman microscope in a unique approach that integrates single-cell Raman metabolic fingerprinting with D_2_O labelling and principal component analysis (PCA) to rapidly and directly detect *H. pylori* resistance to both clarithromycin and levofloxacin. While previous studies have shown the potential of Raman spectroscopy for *H. pylori* detection and antimicrobial susceptibility testing against specific drugs, our method offers a novel, combined strategy. By focusing on the metabolic activity of individual cells via D_2_O incorporation, we provide a rapid, phenotype-based assessment of resistance that complements traditional growth-based assays. This study aims to leverage the rapid, non-invasive and specific advantages of Raman spectroscopy to delve deeper into the detection of *H. pylori* and its resistance mechanisms, thereby providing a theoretical foundation and supplementary data.

## Methods

### Culture-based MICs determination of *H. pylori*

*H. pylori* strain was thawed from a −80 °C freezer, and 50 μl of the bacterial suspension was evenly spread onto Columbia blood agar plates. These plates were then incubated at 37 °C under microaerophilic conditions for 3 days. Bacterial suspensions were adjusted to a McFarland 2.0 standard and streaked onto Columbia agar plates containing serial dilutions of antibiotics. After incubation at 37 °C for 3 days, the MICs of *H. pylori* for levofloxacin and clarithromycin were determined. According to Clinical and Laboratory Standards Institute (CLSI) guidelines guidelines, the breakpoint concentration for clarithromycin is 1.0 μg ml^−1^. However, CLSI does not provide breakpoint standards for levofloxacin. Therefore, based on existing literature, a breakpoint concentration of 1.0 µg ml^−1^ for levofloxacin was adopted for *H. pylori* in this study [19]. In a previous study, 20 clinical *H. pylori* isolates were evaluated for their resistance phenotypes using an *H. pylori* antimicrobial susceptibility testing kit (agar dilution method). Two strains (*H. pylori*-YY and *H. pylori*-ZLR) were selected based on their resistance profiles against clarithromycin and levofloxacin. The results showed that *H. pylori*-YY was sensitive to both antibiotics, with MICs below 0.5 µg ml^−1^; *H. pylori*-ZLR was resistant to both antibiotics, with MICs above 1.0 µg ml^−1^. The MICs for these two antibiotics, determined using this culture-based method, will serve as a reference for subsequent investigations of *H. pylori* antibiotic resistance using Raman metabolic fingerprinting technology.

### PCA of Raman metabolic fingerprint for drug resistance correlation

Bacterial cultures in the logarithmic growth phase were adjusted to a McFarland 2.0 standard. Subsequently, 40% D_2_O and a range of antibiotic concentrations (0, 0.5, 1.0, 2.0 µg ml^−1^) were added, and the cultures were incubated under microaerophilic conditions for 4 h. Bacteria were then harvested by centrifugation at 170 r.p.m. for 4 min, washed thrice with PBS and resuspended in a small volume of PBS. Raman spectra were collected using an Alpha300R Raman microscope (WITec, Germany), which operated with a 532 nm excitation wavelength, a 100× objective lens, a laser power of 7 mW, a 600 lines per millimetre grating and an integration time of 18 s. The spectral resolution was 2 cm⁻¹, and the wavenumber range was 280–3,500 cm⁻¹. A total of ~100 bacterial spectra were collected for each sample. Data preprocessing was performed on the 100 individual spectra, and the mean value and sd were calculated. Using R version 3.6.3, PCA analysis was performed on the spectra within the 400–1,800 cm⁻¹ range using the PCA function from the Modern Applied Statistics with S and caret packages. Two-dimensional (2D) PCA score plots were generated using the plot function to visualize similarities and differences among the spectra of various treatment groups. The MIC was defined as the lowest concentration at which the Raman spectral cluster of the treated group was distinctly separated from that of the control group. Each cluster was represented by the mean ± 2 sd, indicating a 95% confidence level. Non-overlapping clusters were considered significantly different (*P*<0.05). The MIC values of clarithromycin and levofloxacin for each strain, as determined by the aforementioned method, were compared with those obtained from the agar dilution reference standard to evaluate the accuracy of the Raman spectroscopic method in determining the MICs of the three antibiotics.

### Raman metabolic fingerprint analysis of *H. pylori* resistance mechanisms

Two *H. pylori* strains, one clarithromycin-sensitive and one clarithromycin-resistant, were selected. Strains were incubated with 0, 0.5 µg ml^−1^ (sensitive breakpoint concentration), and 1 µg ml^−1^ (resistant breakpoint concentration) of clarithromycin for 4 h. Post-incubation, Raman spectra were acquired. The distribution of Raman spectra along the principal components (PCs) was visualized using histograms to identify Raman shifts that differentiated the spectral profiles of sensitive and resistant strains. The biologically active molecules associated with these distinctive Raman shifts were analysed to explore their potential roles in antibiotic resistance mechanisms.

### Exploring the incubation time of D_2_O with *H. pylori*

Bacterial cultures in the logarithmic growth phase were adjusted to a McFarland 2.0 standard and inoculated into a 24-well plate (1 ml system containing 40% D_2_O). Cultures were incubated at 37 °C under microaerophilic conditions for 0, 4, 6 and 8 h. Bacteria were harvested, washed thrice with sterile water at each time point and prepared as Raman bacterial suspensions. Raman spectra were acquired from 20 to 30 bacteria per sample, with one spectrum recorded from the central position of each bacterium. Raw spectral data were preprocessed to remove cosmic-ray interference, and the 1,800–3,400 cm⁻¹ spectral range was extracted. Baseline correction was performed using a linear method (order 6, noise points 98, maximum points 10), followed by area normalization. The C-D peak (2,040–2,300 cm⁻¹) and C-H peak (2,800–3,100 cm⁻¹) regions were analysed. Using R version 3.6.3, the C-D/(C-H + C-D) stoichiometric ratio was calculated for each sample group. Mean spectra, bar charts and box plots were generated. The shortest incubation time required for the detection of the C-D peak was determined.


$$CD_{norm}=CD_{i}-CD_{nc}/CD_{pc}-CD_{nc}$$

CD_norm_ represents the normalized C-D value; CD_i_ represents the actual C-D value; CD_nc_ represents the C-D value of the negative cell control; and CD_pc_ represents the C-D value of the positive cell control.

### Exploration of the antibiotic incubation system

Two experimental groups were designed as follows: in the first group, *H. pylori* was incubated with the antibiotic for 2 h prior to the addition of D_2_O, followed by incubation, sample collection and Raman spectrum acquisition. In the second group, *H. pylori* was incubated simultaneously with the antibiotic and D_2_O, followed by sample collection and Raman spectrum acquisition. The heterogeneity of D-substitution was quantified by calculating the sd of the C-D ratio for individual cells. A decrease in the C-D peak intensity to below 75% of the (positive–negative) value was considered indicative of the effective inhibitory concentration of the antibiotic, representing the MIC determined by the Raman-deuterium labelling method. If the MIC values obtained from the two measurements differed by a factor of 2 or less, the data were considered valid, with the error remaining within an acceptable range. Compared with the agar dilution method, the C-D ratio method, which exhibited high consistency, was selected as the screening method for determining MICs.

### Criteria for assessing *H. pylori* resistance

After establishing the antibiotic incubation system, individual strains of *H. pylori* were incubated with three different antibiotic concentration gradients in the presence of D_2_O. Raman metabolic fingerprints were collected, and the raw spectral data were preprocessed to remove cosmic-ray interference and to extract the 2,800–3,100 cm⁻¹ spectral region. Baseline correction was then applied. At least 20 valid spectra were acquired for each treatment group. The preprocessed spectra were analysed using R 3.6.3 to calculate the C-D/(C-H + C-D) ratio. Subsequently, mean spectra, bar plots and box plots were generated. The concentration at which the intensity of the C-D peak decreased to less than 75% of the (positive–negative) control was defined as the MIC of the antibiotic against the bacteria, as determined by Raman–heavy-water labelling. Finally, the results of *H. pylori* resistance analysis based on the C-D intensity of Raman metabolic maps were compared with those obtained from traditional gold-standard methods.

## Results

### Single-cell Raman spectroscopy and analysis of *H. pylori*

**Fig. 1.**
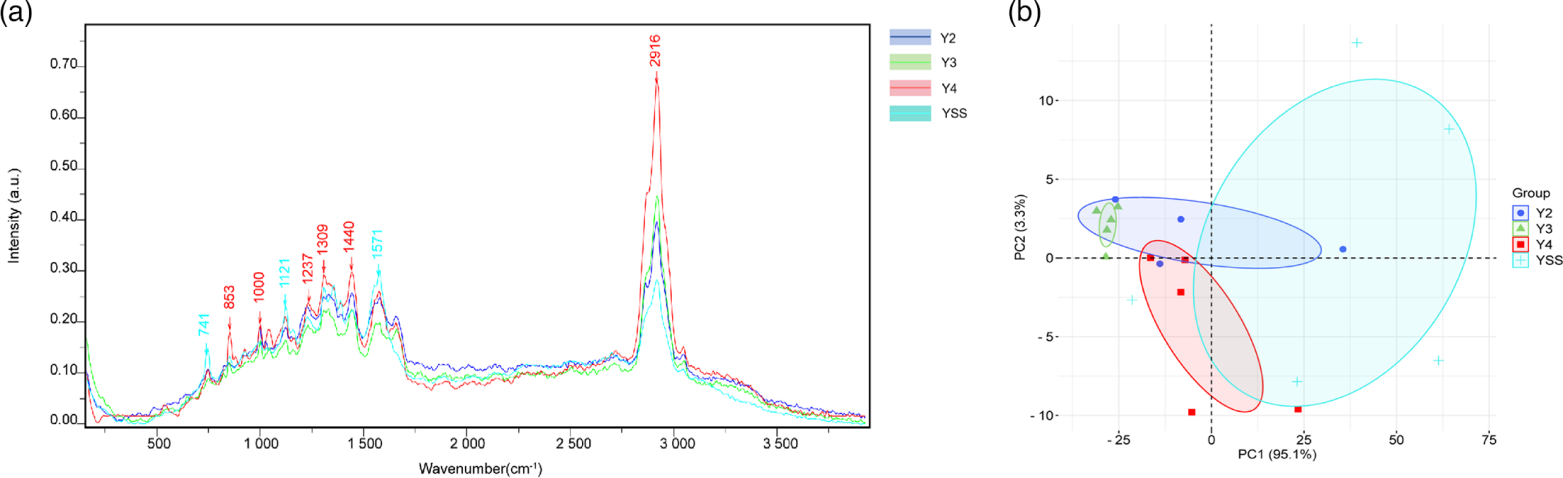
Single-cell Raman spectroscopy analysis of *H. pylori* strains. (**a**) Averaged, normalized Raman spectra of four *H. pylori* strains (YSS, Y2, Y3 and Y4). Data were baseline-corrected and area-normalized. (**b**) PCA scores plot based on the first two PCs. Different colours represent different strains, and ellipses represent 95 % confidence intervals.

After background subtraction and normalization, we analysed the single-cell Raman spectra collected from four *H. pylori* strains (YSS, Y2, Y3 and Y4). The Raman spectral range was ~500–3,500 cm⁻¹. As shown in Fig. 1a, the single-cell Raman spectra of these four strains shared nine characteristic peaks. Table 1 lists the wavenumbers of the nine significantly different characteristic peaks and their corresponding biomolecular assignments. Raman spectra revealed a characteristic peak at 741 cm⁻¹ in all *H. pylori* strains, indicating the presence of methionine in the proteins of these strains and a similar local structural environment [20]. This could be related to the initiating role of methionine in protein synthesis, its unique side-chain structure and the conserved protein structure of *H. pylori* to adapt to the gastric environment. The characteristic peaks observed at 853 and 1,000 cm⁻¹ in the Raman spectra are mainly attributed to the vibrations of the phenyl ring structure of aromatic amino acids (such as phenylalanine, tyrosine and tryptophan) in bacterial proteins. These amino acids not only contribute to protein structural stability but may also participate in protein–protein interactions and play important roles in the physiological functions of *H. pylori* [21]. Characteristic peaks of proteins were observed at 1,121 and 1,237 cm⁻¹, primarily attributed to the vibrations of specific chemical bonds within the protein molecule. Proteins are biological macromolecules composed of amino acids linked together by peptide bonds, resulting in C-N stretching and coupled vibrations typically appearing in the 1,200–1,300 cm⁻¹ region [22]. Additionally, the secondary structure of proteins (e.g. α-helices and β-sheets) can influence Raman spectra, causing shifts and intensity changes in the bands due to alterations in the microenvironment of the peptide bond. Peaks at 1,309 and 1,440 cm⁻¹ indicated the presence of both proteins and lipids. The signal at 1,309 cm⁻¹ primarily originated from the overlap of C-N stretching vibrations in the peptide bond and bending vibrations of the methylene group (CH_₂_) group in amino acid side chains, while the signal at 1,440 cm⁻¹ corresponded to the scissoring vibration of CH_₂_, mainly from the CH_₂_ groups in the fatty acid chains of phospholipid molecules in the cell membrane [23]. Overall, these characteristic peaks reflect the abundant protein and lipid content in *H. pylori* cells, as well as the vibrational features of specific chemical bonds. The peak at 1,571 cm⁻¹ primarily originates from the overlapping C=C stretching vibrations of the aromatic ring structures in aromatic amino acids and nucleic acids. The side chains of aromatic amino acids contain benzene rings, whose C=C stretching vibrations typically occur in the 1,600–1,580 cm⁻¹ range, while the bases of nucleic acids (such as purines and pyrimidines) also possess aromatic ring structures that can give rise to related vibrations in the same frequency range [24]. The Raman spectrum of *H. pylori* exhibits a characteristic peak at 2,916 cm⁻¹, mainly reflecting the C-H stretching vibrations of C-H bonds in lipids, proteins (side chains) and nucleic acids. This vibration typically occurs in the 2,800–3,000 cm⁻¹ range, and the peak formation is likely due to the combined contributions of CH_₂_ and CH_₃_ groups in the fatty acid chains of lipid molecules, amino acid side chains in proteins, and bases and sugars in nucleic acid molecules [25].

**Table 1.** Biological attribution of Raman wavenumbers

Raman wavenumber (cm^−1^)	Biomolecule assignment	References
741	Proteins (containing methionine)	[20]
853	Aromatic amino acids (tryptophan, tyrosine, phenylalanine)	[40]
1,000	Aromatic amino acids (tryptophan, tyrosine, phenylalanine)	[41]
1,121	Proteins	[42,43]
1,237	Proteins	[42,43]
1,309	Proteins, lipids	[44,45]
1,440	Lipids, proteins (side chains)	[44,45]
1,571	Aromatic amino acids (tryptophan, tyrosine, phenylalanine), nucleic acids (purines, pyrimidines)	[46,47]
2,916	Lipids, proteins (side chains), nucleic acids	[48]

As shown in Fig. 1b, PCA analysis of single-cell Raman spectra from four *H. pylori* strains revealed a clear clustering trend. Strains YSS, Y2 and Y4 clustered closely together, suggesting that they may belong to similar *H. pylori* subtypes or genotypes, or may have grown in similar environments or exhibit similar metabolic characteristics. This indicates a high degree of similarity in their cellular composition and molecular structure. In contrast, strain Y3 clustered distinctly from the other three strains, showing significant differences. These differences may be attributed to several factors: first, different *H. pylori* genotypes have distinct genome sequences and protein expression profiles; second, even within the same genotype, *H. pylori* strains may exhibit phenotypic differences due to adaptation to different host environments or other factors, leading to alterations in Raman spectral features.

Single-cell Raman spectroscopy, in conjunction with PCA, preliminarily demonstrates its potential for discerning variations in the structure and abundance of key biomolecular components, including proteins, nucleic acids and lipids, within *H. pylori*. This approach may also offer a means of differentiating between distinct strains. These initial observations suggest that Raman spectroscopy holds promise as a rapid, non-invasive and high-throughput methodology for *H. pylori* detection and typing, potentially contributing novel insights into the mechanisms underlying *H. pylori* pathogenesis.

### 2D PCA method for analysing the correlation with *H. pylori* resistance

**Fig. 2.**
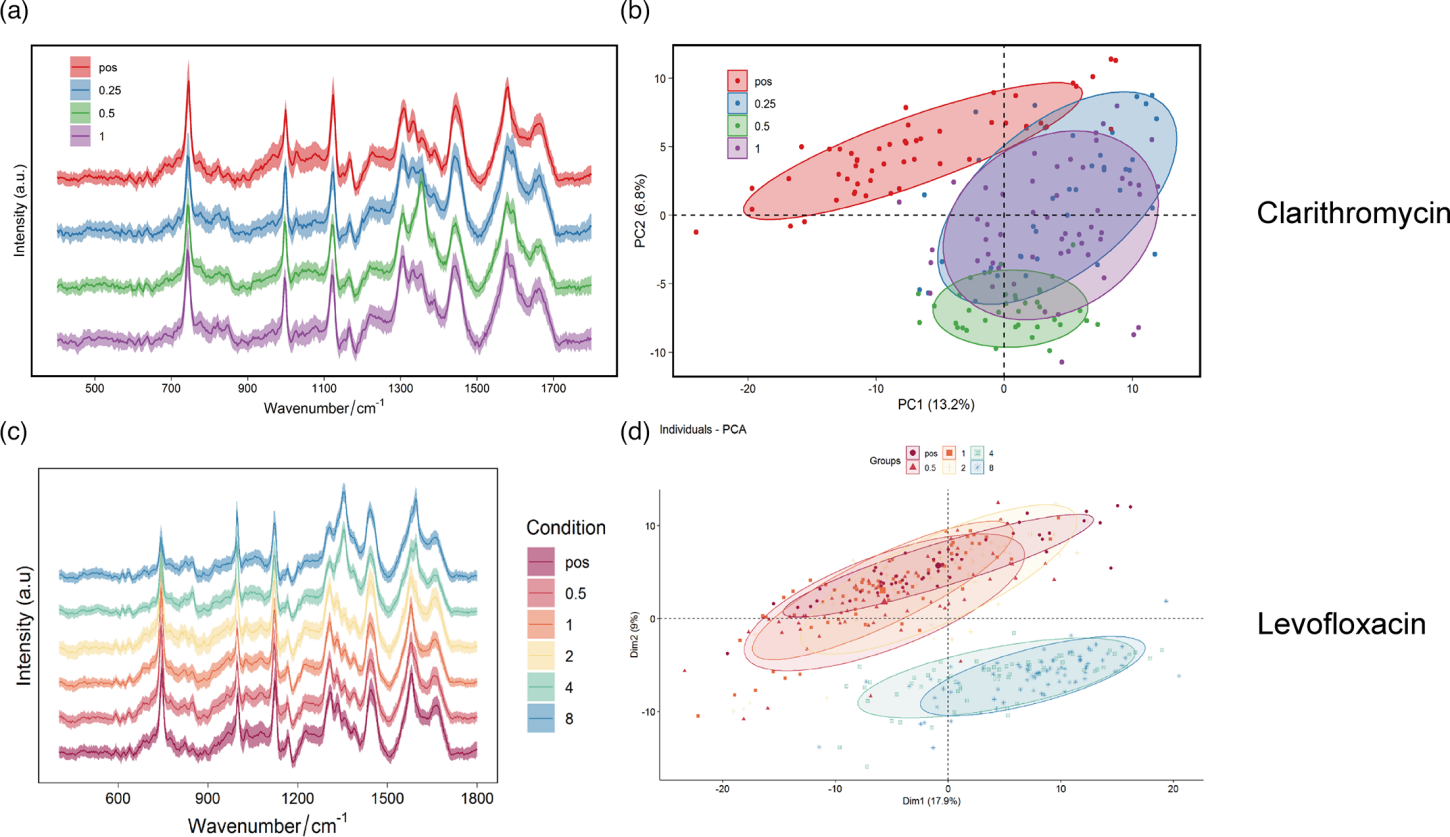
PCA analysis of *H. pylori*-YY resistance to clarithromycin and levofloxacin. (**a, c**) Raman spectral fingerprints of *H. pylori*-YY treated with different concentrations of clarithromycin (**a**) and levofloxacin (**c**). (**b, d**) 2D PCA score plots illustrating *H. pylori*-YY responses to clarithromycin (**b**) and levofloxacin (**d**) at various concentrations. Note: The ‘pos’ group represents the positive control, where the strain was cultured without antibiotic, serving as a reference for normal growth conditions and the corresponding Raman spectral fingerprint. Dim1 and Dim2 represent the first and second principal components, respectively, which capture the largest variance in the Raman spectral data.

Fig. 2a and c display the Raman spectra of *H. pylori*-YY after treatment with clarithromycin and levofloxacin, respectively . shown in the PCA of Raman spectra in Fig. 2b, the samples treated with 0.25, 0.5 and 1 µg ml^−1^ clarithromycin were clearly separated from the control group, indicating that the Raman spectral characteristics of *H. pylori*-YY changed significantly under the action of clarithromycin. Combined with the phenotypic drug sensitivity test (the agar dilution method detected that the MIC of *H. pylori*-YY to clarithromycin was lower than 0.50 µg ml^−1^), this suggests that the MIC of *H. pylori*-YY to clarithromycin may be 0.25 µg ml^−1^, indicating its sensitivity to clarithromycin. As shown in Fig. 2d, in the PCA analysis, the clustering patterns of the samples treated with 0.5, 1.0 and 2.0 µg ml^−1^ levofloxacin were difficult to distinguish from the untreated control group, indicating that the Raman spectral characteristics of *H. pylori*-YY under these concentrations of levofloxacin were not significantly different from the control group. However, the samples treated with 4.0 and 8.0 µg ml^−1^ were clearly separated from the control group, indicating that the Raman spectral characteristics of *H. pylori* changed significantly under high concentrations of levofloxacin. Combined with the phenotypic drug sensitivity test (the agar dilution method detected that the MIC of *H. pylori* to levofloxacin was lower than 0.50 µg ml^−1^), it shows that *H. pylori* is sensitive to levofloxacin. However, the Raman spectroscopy combined with PCA analysis showed that the Raman spectral characteristics of *H. pylori*-YY only changed significantly under high concentrations of levofloxacin (4.0 µg ml^−1^ or higher), suggesting that *H. pylori*-YY may have some kind of resistance mechanism to levofloxacin. Raman spectroscopy combined with PCA to detect the drug resistance phenotype of *H. pylori*-YY to clarithromycin is consistent with the results of the drug sensitivity test, while the drug resistance phenotype to levofloxacin is inconsistent with the results of the drug sensitivity test. The principles of Raman spectroscopy combined with PCA analysis and traditional drug sensitivity tests to detect bacterial drug resistance are different. The former detects the overall molecular composition and metabolic changes of bacteria under the action of drugs, while the latter mainly detects the growth inhibition of bacteria. Therefore, there may be differences in the results of the two methods, which may be because although bacterial growth is inhibited under low concentrations of drugs, the changes at the molecular level are not sufficient to be detected by Raman spectroscopy.

In the context of the PCA analysis shown in Fig. 1b, the separation of strains YSS, Y2, Y3 and Y4 suggests that single-cell Raman spectroscopy has the potential to differentiate between *H. pylori* strains. However, we acknowledge that, without further validation, using techniques such as WGS, fluorescence *in situ* hybridization or machine-learning-based classification models, the application of this method for reliable species or strain typing in complex, multi-microbe samples remains a significant challenge. This study serves as a foundational step, and future work is needed to develop a comprehensive approach that integrates Raman-based phenotyping with genotypic data for accurate and robust strain identification in clinical settings.

### D_2_O can participate in the metabolism of *H. pylori* (in the silent zone)

**Fig. 3.**
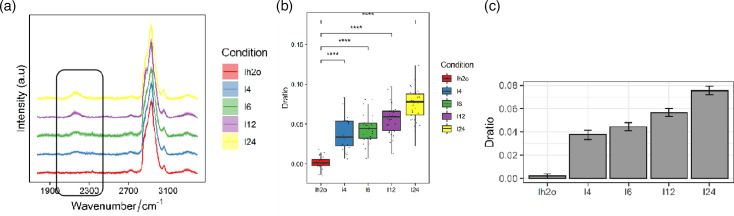
Detection of C-D stretching vibration following D_2_O supplementation. (**a**) Raman spectra of *H. pylori* in the silent region collected at different incubation time points. (**b**) Box plot showing the C-D/(C-D + C-H) ratio at different incubation times. (**c**) Bar graph illustrating the C-D/(C-D + C-H) ratio at different incubation times.

To investigate the involvement of D_2_O in *H. pylori* metabolism, 50% D_2_O was added to the liquid culture medium of *H. pylori* strains. Bacterial cultures were harvested at 4, 6, 12 and 24 h post-inoculation, prepared for Raman spectroscopy, and their spectra were acquired. The C-D peak intensity within the silent region (2,040–2,300 cm^−1^) was analysed. Our results showed that the C-D peak intensity increased significantly over time, suggesting a time-dependent accumulation of deuterium incorporation. As shown in Fig. 3, the C-D/(C-D + C-H) ratio in the experimental group was significantly higher than that in the control group starting from 4 h of incubation, indicating a rapid enrichment of deuterium atoms in *H. pylori*. This result clearly demonstrates that D_2_O can efficiently penetrate into bacterial cells and participate in cellular metabolic processes even within a short time. Thus, a 4 h incubation time was chosen for subsequent experiments.

### The intensity of the C-D peak can indicate *H. pylori* resistance

**Fig. 4.**
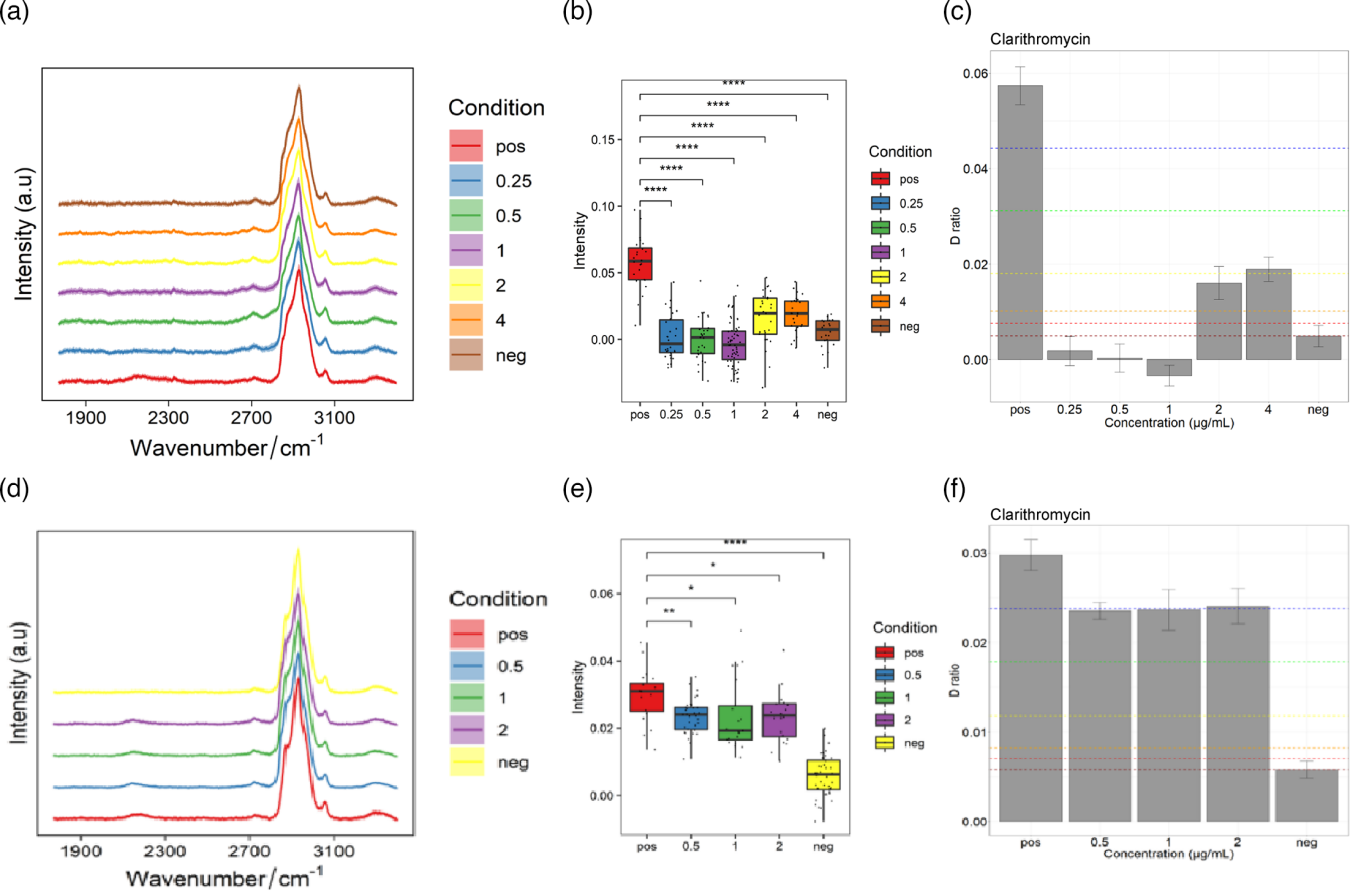
Correlation between clarithromycin resistance and C-D peak ratios in the silent region. (**a, d**) Raman spectra of *H. pylori*-YY (**a**) and *H. pylori*-ZLR (**d**) in the silent region under various clarithromycin concentrations. (**b, e**) Box plots illustrating the C-D/(C-D + C-H) ratio for *H. pylori*-YY (**b**) and *H. pylori*-ZLR (**e**) at different clarithromycin concentrations. (**c, f**) Bar graphs showing the C-D/(C-D + C-H) ratio for *H. pylori*-YY (**c**) and *H. pylori*-ZLR (**f**) at different clarithromycin concentrations. The ‘pos’ group represents the positive control, where the strain was cultured without antibiotic, while the ‘neg’ group represents the negative control, where the strain was cultured with inhibitory concentrations of the antibiotic. Note: In the spectra, the 2,040–2,300 cm⁻¹ region corresponds to the deuterium peak, while the 2,800–3,000 cm⁻¹ region represents the C-H stretching vibration. In the bar graphs, the horizontal lines from top to bottom indicate the 75th, 50th, 25th, 10th, 5th, and 0th percentiles, respectively.

*H. pylori-*YY and *H. pylori-*ZLR were incubated with various concentrations of clarithromycin for 4 h. Bacterial cells were harvested, and Raman spectra were acquired. The C-D/(C-D + C-H) ratio was calculated to assess the inhibitory effects of clarithromycin. A decrease in the C-D peak intensity to below 75% of the positive–negative value was considered indicative of effective antibiotic inhibition, thus defining the Raman-deuterium labelling-based MIC (Fig. 4). The MIC of *H. pylori-*YY to clarithromycin was determined to be ≤0.25 µg ml^−1^, below the CLSI breakpoint of 1.0 µg ml^−1^, indicating susceptibility. In contrast, the MIC of *H. pylori-*ZLR was >2.0 µg ml^−1^, above the breakpoint, suggesting resistance. These findings were consistent with the phenotypes obtained using the agar dilution method.

**Fig. 5.**
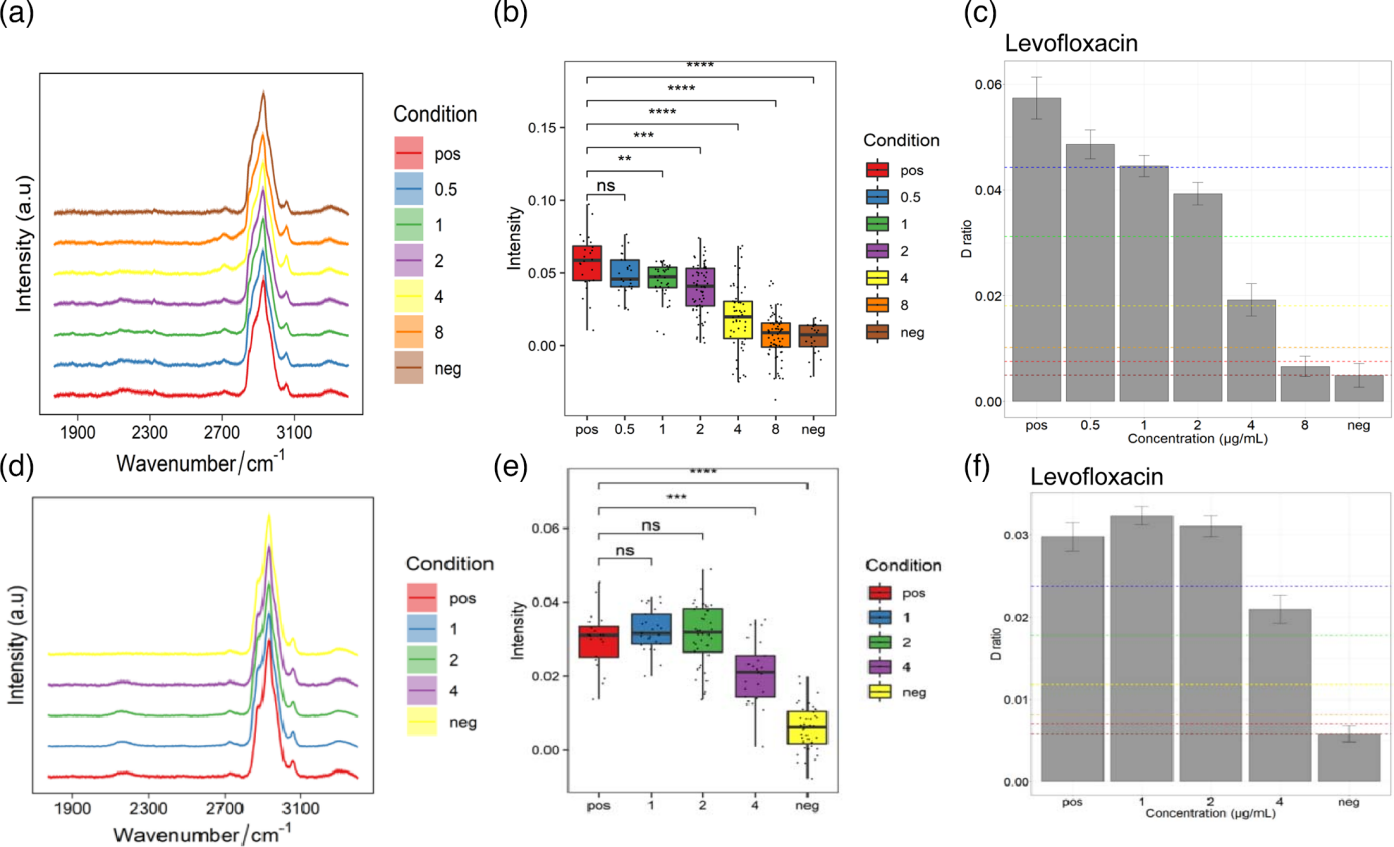
Correlation between levofloxacin resistance and C-D peak ratios in the silent region. (**a, d**) Raman spectra of *H. pylori-*YY (**a**) and *H. pylori-*ZLR (**d**) in the silent region under various levofloxacin concentrations. (**b, e**) Box plots illustrating the C-D/(C-D + C-H) ratio for *H. pylori-*YY (**b**) and *H. pylori-*ZLR (**e**) at different levofloxacin concentrations. (**c, f**) Bar graphs showing the C-D/(C-D + C-H) ratio for *H. pylori-*YY (**c**) and *H. pylori-*ZLR (**f**) at different levofloxacin concentrations. The ‘pos’ group represents the positive control, where the strain was cultured without antibiotic, while the ‘neg’ group represents the negative control, where the strain was cultured with inhibitory concentrations of the antibiotic.

Using the same methodology, *H. pylori-*YY and *H. pylori-*ZLR were incubated with various concentrations of levofloxacin for 4 h. Post-incubation, bacterial cells were harvested, and Raman spectra were acquired. The C-D/(C-D + C-H) ratio was calculated to determine the MIC. As depicted in Fig. 5, the MICs of *H. pylori-*YY and *H. pylori-*ZLR to levofloxacin were 2.0 and 4.0 µg ml^−1^, respectively. A comparison with previous agar dilution results revealed an inconsistency in the resistance phenotype of *H. pylori-*YY: susceptible by agar dilution but resistant by Raman-deuterium labelling. However, the resistance phenotype of *H. pylori-*ZLR remained consistent, classified as resistant by both methods. This discrepancy highlights a key difference between the two detection principles. The agar dilution method primarily measures the macroscopic outcome of bacterial growth inhibition, whereas the Raman-deuterium labelling method directly probes the metabolic activity of individual cells. This suggests that, even at low, sub-inhibitory concentrations of levofloxacin, the metabolic processes of the *H. pylori*-YY strain were significantly altered, although its overall growth was not completely halted. This observation indicates a potential metabolic-level resistance mechanism that may not be fully captured by traditional growth-based assays.

### Screening biomacromolecules associated with clarithromycin resistance

**Fig. 6.**
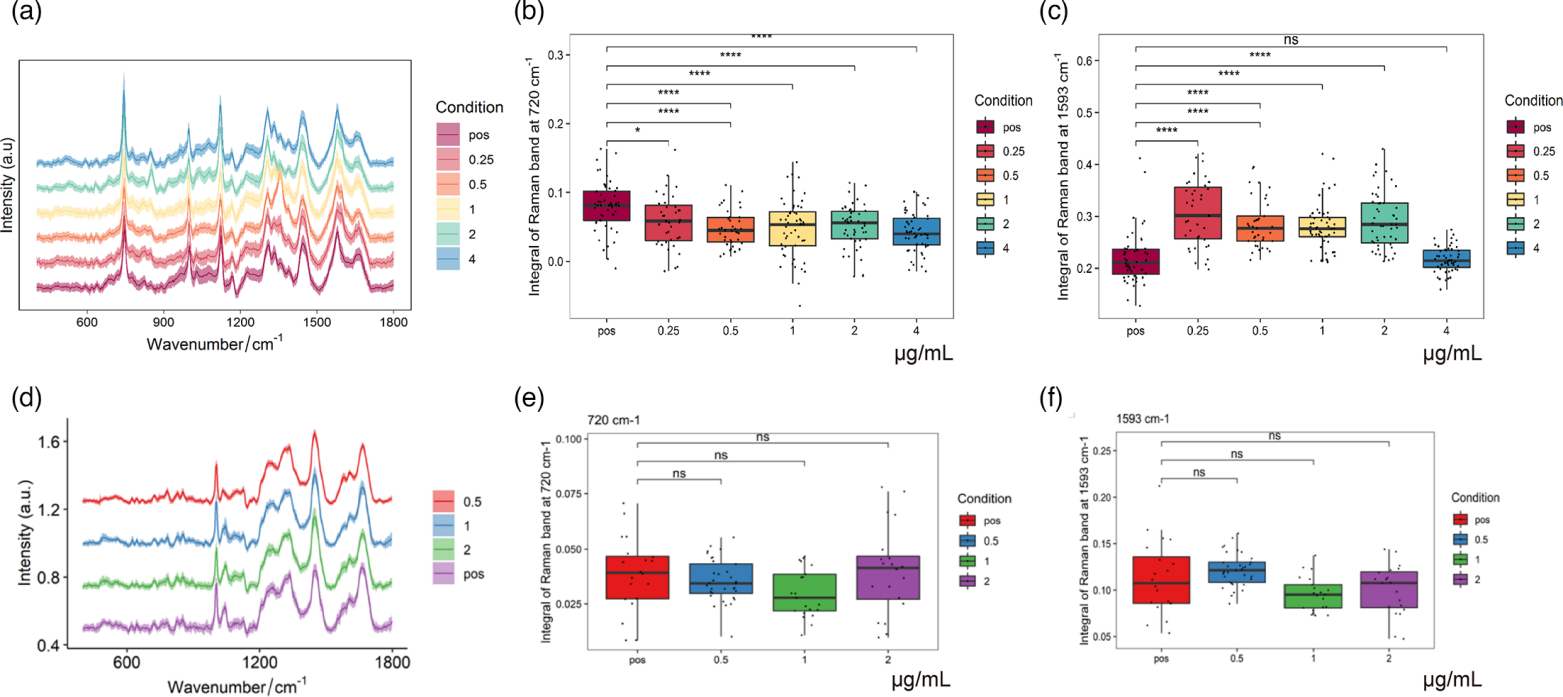
Statistical analysis of the fingerprint region at 720 and 1,593 cm⁻¹ in *H. pylori-*YY and *H. pylori-*ZLR following clarithromycin treatment. (**a, d**) Raman fingerprint spectra of *H. pylori-*YY (**a**) and *H. pylori-*ZLR (**d**) obtained after treatment with various clarithromycin concentrations. (**b, e**) Statistical analysis of the extracted spectra at 720 cm⁻¹ for *H. pylori-*YY (**b**) and *H. pylori-*ZLR (**e**). (**c, f**) Statistical analysis of the extracted spectra at 1,593 cm⁻¹ for *H. pylori-*YY (**c**) and *H. pylori-*ZLR (**f**). Note: The ‘pos’ group represents the positive control, where the strain was cultured without antibiotic, serving as a reference for normal growth conditions and the corresponding Raman spectral fingerprint.

After clarithromycin treatment, compared with the control group, the 720 cm^−1^ peak (adenine) of *H. pylori*-YY (susceptible strain) decreased significantly at a concentration of 0.25 µg ml^−1^ (Fig. 6). This suggests that clarithromycin effectively inhibited nucleic acid synthesis in the susceptible strain, leading to a decrease in adenine content [26]. Conversely, at the same concentration, the 1,593 cm^−1^ peak (in-plane bending mode of phenylalanine C=C) significantly increased, which may be related to enhanced protein synthesis or cell wall structural modifications in the bacteria in response to antibiotic stress [27]. In contrast, no significant changes were observed in the 720 and 1,593 cm^−1^ peaks of *H. pylori*-ZLR (resistant strain), suggesting that the resistant strain was less sensitive to clarithromycin treatment, and its nucleic acid and protein synthesis metabolic processes were not significantly affected. These results indicate that the 720 and 1,593 cm^−1^ peaks may serve as potential biomarkers for studying clarithromycin resistance mechanisms.

## Discussion

Due to factors such as *H. pylori* resistance, eradication strategies for *H. pylori* vary across countries, with most adopting empirical therapy that typically combines two antibiotics [28,32]. Long-term antibiotic use can lead to adverse effects, such as diarrhoea and gut dysbiosis, consequently impacting overall health and potentially increasing the risk of developing diseases like diabetes and Alzheimer’s [33,35]. Therefore, the precise use of medication during initial treatment to improve eradication efficacy is crucial for effectively controlling *H. pylori* and reducing its resistance. One of the primary causes of *H. pylori* eradication failure is antibiotic resistance, particularly to clarithromycin and levofloxacin [36,37]. Conventional phenotypic susceptibility testing for *H. pylori* is time-consuming, labour-intensive and highly susceptible to human error, making it inadequate for clinical needs [38]. To improve *H. pylori* eradication rates, a rapid and accurate resistance detection method is urgently needed.

In this study, we employed single-cell Raman spectroscopy to delve into the antibiotic resistance of *H. pylori*. By utilizing 2D PCA and C-D ratio analysis, we found that D_2_O could reflect the metabolic activity of *H. pylori*, and the Raman spectra of bacteria exhibited significant differences under various antibiotic treatments, allowing us to distinguish between antibiotic-sensitive and -resistant strains against clarithromycin and levofloxacin. Our findings suggest that Raman spectroscopy holds great promise as a rapid and non-destructive method for detecting bacterial antibiotic resistance. Notably, previous studies have preliminarily revealed the potential of Raman spectroscopy in exploring the antibiotic resistance of *H. pylori* [18,39]. Our research further confirms the broad application prospects of Raman spectroscopy in bacterial antibiotic resistance studies. By comparing the Raman spectral characteristics of different antibiotics, we can gain deeper insights into bacterial antibiotic resistance mechanisms and provide a theoretical basis for the development of novel antibiotics.

While this study successfully demonstrates the ability of single-cell Raman spectroscopy to rapidly detect antibiotic resistance phenotypes, we acknowledge that it provides a high-level metabolic fingerprint rather than a complete picture of the precise biochemical pathways involved. For example, the observed changes in peaks related to nucleic acids and proteins offer strong evidence of altered synthesis in response to antibiotic stress. These spectral shifts serve as a basis for hypothesis generation, and future research will be dedicated to a more in-depth investigation of these pathways. This could involve combining Raman spectroscopy with other omics techniques, such as transcriptomics or metabolomics, to correlate specific spectral changes with gene expression profiles or metabolite concentrations. Such an integrated approach would allow us to move beyond phenotypic observation towards a more detailed understanding of the dynamic metabolic responses that drive antibiotic resistance in *H. pylori*.

The observed discrepancy in levofloxacin susceptibility for the *H. pylori-*YY strain, where traditional agar dilution indicated susceptibility while Raman-deuterium labelling suggested resistance, is a critical finding that warrants further discussion. This inconsistency underscores the fundamental difference between these two methods. Traditional phenotypic testing relies on a macroscopic assessment of bacterial growth over a prolonged period (e.g. 3 days), while our Raman-based approach provides a rapid, single-cell, metabolic-level snapshot of the bacterial response to an antibiotic (within 4 h). The Raman-deuterium labelling method detects any significant reduction in D_2_O incorporation, which is a direct proxy for metabolic activity, irrespective of whether the bacteria are fully inhibited from multiplying. Therefore, a strain may be metabolically ‘stressed’ by a sub-inhibitory concentration of an antibiotic, leading to a decreased C-D ratio and a classification of resistance by our method, even if it could eventually form colonies on agar. This suggests that our technique might be more sensitive in detecting early-stage or sub-lethal antibiotic effects. Future research will explore this phenomenon in more detail, potentially combining Raman spectroscopy with real-time growth curves and molecular analysis to better understand these genotype–phenotype inconsistencies and the dynamic metabolic shifts that underlie antibiotic tolerance and resistance.

While our current methodology focuses on isolated *H. pylori* strains to establish the fundamental principles of resistance detection via single-cell Raman spectroscopy, we acknowledge the practical challenge posed by mixed bacterial populations and potential contaminants in clinical samples. The unique spectral fingerprints of different microbial species, and indeed host cells, could introduce interference. However, the single-cell nature of our Raman spectroscopy approach offers a distinct advantage in this regard. By precisely targeting individual bacterial cells, we can potentially mitigate the impact of confounding signals from other micro-organisms or host material present in a heterogeneous sample. Future developments will explore advanced spectral unmixing algorithms and pre-analytical sample preparation strategies (e.g. specific cell enrichment or microfluidic isolation) to enhance the purity of *H. pylori* cell analysis in real-world clinical specimens. This will be a critical step towards translating our findings into a robust diagnostic tool applicable to complex patient samples.

Furthermore, the significant reduction in detection time, from the traditional 3-day culture-based methods to a rapid 4–6 h turnaround time using our Raman spectroscopy approach, carries substantial clinical significance. Current clinical practice often relies on empirical therapy while awaiting susceptibility results, a process that can take several days. If the chosen empirical regimen fails due to resistance, patients may endure prolonged discomfort and face increased risks associated with repeated treatment cycles. By providing a rapid and accurate resistance profile within hours, our method enables clinicians to prescribe personalized, targeted therapy during the patient’s initial visit. This shift from empirical to precision medicine is crucial for improving first-line eradication success rates, minimizing patient burden and reducing the unnecessary use of broad-spectrum antibiotics, thereby helping to curb the overall rise of antibiotic resistance. This temporal advantage translates directly into improved patient outcomes and more efficient use of healthcare resources.

## Conclusion

Our study preliminarily validated the feasibility of single-cell Raman spectroscopy in differentiating *H. pylori* antibiotic resistance and demonstrated its advantages of being rapid, non-invasive and information-rich, with the potential to improve *H. pylori* eradication rates. However, the sample size of this study was relatively small, and the exploration of resistance mechanisms was not deep enough. In the future, we will expand the sample size, delve deeper into the expression changes of resistance-related genes and develop a high-throughput, portable and automated detection platform based on Raman spectroscopy. Meanwhile, by combining multi-omics data, such as genomics and proteomics, we will comprehensively analyse the molecular mechanisms of *H. pylori* resistance, providing a rapid and accurate resistance detection method for clinical applications to guide personalized treatment.

## Abbreviations

CLSI: Clinical and Laboratory Standards Institute
2D: two-dimensional
D_2_O: deuterium oxide
PC: principal component
PCA: principal component analysis
SERS: surface-enhanced Raman spectroscopy
WGS: whole-genome sequencing

## References

[1] Tshibangu-Kabamba E, Yamaoka Y (2021). Helicobacter pylori infection and antibiotic resistance - from biology to clinical implications. Nat Rev Gastroenterol Hepatol.

[2] Malfertheiner P, Megraud F, Rokkas T, Gisbert JP, Liou J-M (2022). Management of *Helicobacter pylori* infection: the maastricht VI/Florence consensus report. Gut.

[3] Sugano K, Tack J, Kuipers EJ, Graham DY, El-Omar EM (2015). Kyoto global consensus report on *Helicobacter pylori* gastritis. Gut.

[4] Liu WZ, Xie Y, Liu H, Cheng H, Zeng Z (2017). Fifth chinese national consensus report on the management of *Helicobacter pylori* infection. Chin J Dig.

[5] Jung YS, Kim EH, Park CH (2017). Systematic review with meta-analysis: the efficacy of vonoprazan-based triple therapy on *Helicobacter pylori* eradication. Aliment Pharmacol Ther.

[6] Yan T-L, Gao J-G, Wang J-H, Chen D, Lu C (2020). Current status of *Helicobacter pylori* eradication and risk factors for eradication failure. World J Gastroenterol.

[7] Han X, Yu X, Gao X, Wang X, Tay CY (2023). Quantitative pcr of string‐test collected gastric material: a feasible approach to detect *Helicobacter pylori* and its resistance against clarithromycin and levofloxacin for susceptibility‐guided therapy. Helicobacter.

[8] Huang WE, Griffiths RI, Thompson IP, Bailey MJ, Whiteley AS (2004). Raman microscopic analysis of single microbial cells. Anal Chem.

[9] Saikia D, Jadhav P, Hole AR, Krishna CM, Singh SP (2022). Unraveling the secrets of colistin resistance with label-free raman spectroscopy. Biosensors.

[10] Liu Z, Xue Y, Yang C, Li B, Zhang Y (2023). Rapid identification and drug resistance screening of respiratory pathogens based on single-cell raman spectroscopy. Front Microbiol.

[11] Yi X, Song Y, Xu X, Peng D, Wang J (2021). Development of a Fast Raman-Assisted Antibiotic Susceptibility Test (FRAST) for the antibiotic resistance analysis of clinical urine and blood samples. Anal Chem.

[12] Yang K, Li H-Z, Zhu X, Su J-Q, Ren B (2019). Rapid antibiotic susceptibility testing of pathogenic bacteria using heavy-water-labeled single-cell raman spectroscopy in clinical samples. Anal Chem.

[13] Zhang M, Hong W, Abutaleb NS, Li J, Dong P-T (2020). Rapid determination of antimicrobial susceptibility by stimulated raman scattering imaging of D_2_O metabolic incorporation in a single bacterium. Adv Sci.

[14] Tang J-W, Li F, Liu X, Wang J-T, Xiong X-S (2024). Detection of *Helicobacter pylori* infection in human gastric fluid through surface-enhanced raman spectroscopy coupled with machine learning algorithms. Lab Invest.

[15] Haider SI, Akhtar N, Saleem M, Ahmed S, Nadeem S (2024). Diagnosis of ‘“*Helicobacter pylori* infection of the gastric biopsy”’ by rapid urease test, histopathology and raman spectroscopy. Diagn Microbiol Infect Dis.

[16] Li F, Si Y-T, Tang J-W, Umar Z, Xiong X-S (2024). Rapid profiling of carcinogenic types of *Helicobacter pylori* infection via deep learning analysis of label-free SERS spectra of human serum. Comput Struct Biotechnol J.

[17] Sun L, Liu M, Gong Y, Zhai K, Lv F (2024). Rapid antimicrobial susceptibility test of *Helicobacter pylori* to metronidazole via single‐cell raman spectrometry. Helicobacter.

[18] Liu M, Zhu P, Zhang L, Gong Y, Wang C (2022). Single-cell identification, drug susceptibility test, and whole-genome sequencing of helicobacter pylori directly from gastric biopsy by clinical antimicrobial susceptibility test ramanometry. Clin Chem.

[19] Luzarraga V, Cremniter J, Plouzeau C, Michaud A, Broutin L (2024). In vitro activity of delafloxacin against clinical levofloxacin-resistant *Helicobacter pylori* isolates. J Antimicrob Chemother.

[20] Spratt SJ, Oguchi K, Miura K, Asanuma M, Kosakamoto H (2022). Probing methionine uptake in live cells by deuterium labeling and stimulated raman scattering. J Phys Chem B.

[21] Abady KK, Karpourazar N, Krishnamoorthi A, Li R, Rentzepis PM (2025). Spectroscopic analysis of bacterial photoreactivation. Photochem Photobiol.

[22] Lee T, Lim J, Park K, Lim E-K, Lee J-J (2020). Peptidoglycan-binding protein metamaterials mediated enhanced and selective capturing of gram-positive bacteria and their specific, ultra-sensitive, and reproducible detection via surface-enhanced raman scattering. *ACS Sens*.

[23] Kuhar N, Sil S, Verma T, Umapathy S (2018). Challenges in application of Raman spectroscopy to biology and materials. RSC Adv.

[24] Pezzotti G, Ohgitani E, Fujita Y, Imamura H, Pappone F (2023). Raman fingerprints of SARS-CoV-2 omicron subvariants: molecular roots of virological characteristics and evolutionary directions. ACS Infect Dis.

[25] Blanch EW, Hecht L, Barron LD (2003). Vibrational Raman optical activity of proteins, nucleic acids, and viruses. Methods.

[26] Marques AT, Vítor JMB, Santos A, Oleastro M, Vale FF (2020). Trends in *Helicobacter pylori* resistance to clarithromycin: from phenotypic to genomic approaches. Microb Genom.

[27] Taylor DE (2000). Pathophysiology of antibiotic resistance: clarithromycin. Can J Gastroenterol.

[28] Malfertheiner P, Megraud F, O’Morain CA, Gisbert JP, Kuipers EJ (2017). Management of *Helicobacter pylori* infection-the maastricht V/Florence consensus report. Gut.

[29] Fallone CA, Chiba N, van Zanten SV, Fischbach L, Gisbert JP (2016). The toronto consensus for the treatment of *Helicobacter pylori* infection in adults. Gastroenterology.

[30] Chey WD, Leontiadis GI, Howden CW, Moss SF (2017). ACG clinical guideline: treatment of *Helicobacter pylori* infection. Am J Gastroenterol.

[31] Jung H-K, Kang SJ, Lee YC, Yang H-J, Park S-Y (2021). Evidence-based guidelines for the treatment of *Helicobacter pylori* Infection in Korea 2020. Gut Liver.

[32] Kato M, Ota H, Okuda M, Kikuchi S, Satoh K (2019). Guidelines for the management of *Helicobacter pylori* infection in Japan: 2016 revised edition. Helicobacter.

[33] Liou J-M, Jiang X-T, Chen C-C, Luo J-C, Bair M-J (2023). Second-line levofloxacin-based quadruple therapy versus bismuth-based quadruple therapy for Helicobacter pylori eradication and long-term changes to the gut microbiota and antibiotic resistome: a multicentre, open-label, randomised controlled trial. Lancet Gastroenterol Hepatol.

[34] Gurung M, Li Z, You H, Rodrigues R, Jump DB (2020). Role of gut microbiota in type 2 diabetes pathophysiology. EBioMedicine.

[35] Zhang T, Gao G, Kwok L-Y, Sun Z (2023). Gut microbiome-targeted therapies for Alzheimer’s disease. Gut Microbes.

[36] Yang J-C, Kao JY (2022). Treatment considerations in *Helicobacter pylori* management. Aliment Pharmacol Ther.

[37] Ansari S, Yamaoka Y (2022). *Helicobacter pylori* infection, its laboratory diagnosis, and antimicrobial resistance: a perspective of clinical relevance. Clin Microbiol Rev.

[38] Sun Q, Yuan C, Zhou S, Lu J, Zeng M (2023). *Helicobacter pylori* infection: a dynamic process from diagnosis to treatment. Front Cell Infect Microbiol.

[39] Sun L, Liu M, Gong Y, Zhai K, Lv F (2024). Rapid antimicrobial susceptibility test of Helicobacter pylori to metronidazole via single-cell raman spectrometry. Helicobacter.

[40] Contorno S, Darienzo RE, Tannenbaum R (2021). Evaluation of aromatic amino acids as potential biomarkers in breast cancer by Raman spectroscopy analysis. Sci Rep.

[41] Turk N, Raza A, Wuytens P, Demol H, Daele MV (2020). Waveguide-based surface-enhanced Raman spectroscopy detection of protease activity using non-natural aromatic amino acids. Biomed Opt Express.

[42] Fang C, Tang L (2020). Mapping structural dynamics of proteins with femtosecond stimulated raman spectroscopy. Annu Rev Phys Chem.

[43] Cai L, Fang G, Tang J, Cheng Q, Han X (2022). Label-free surface-enhanced raman spectroscopic analysis of proteins: advances and applications. IJMS.

[44] Benevides JM, Overman SA, Thomas GJ Jr (2004). Raman spectroscopy of proteins. Curr Protoc Protein Sci.

[45] Radwan B, Adamczyk A, Tott S, Czamara K, Kaminska K (2020). Labeled vs. label-free raman imaging of lipids in endothelial cells of various origins. Molecules.

[46] Calderon I, Guerrini L, Alvarez-Puebla RA (2021). Targets and tools: nucleic acids for surface-enhanced raman spectroscopy. Biosensors (Basel).

[47] Hernández B, Pflüger F, Adenier A, Kruglik SG, Ghomi M (2010). Vibrational analysis of amino acids and short peptides in hydrated media. VIII. amino acids with aromatic side chains: l-phenylalanine, l-tyrosine, and l-tryptophan. J Phys Chem B.

[48] Zhang X, Roeffaers MB, Basu S, Daniele JR, Fu D (2012). Label-free live-cell imaging of nucleic acids using stimulated Raman scattering microscopy. Chemphyschem.

